# Multiplexer-based system development for multichannel neurochemical sensing

**DOI:** 10.1007/s13534-026-00554-3

**Published:** 2026-03-02

**Authors:** Haeun Kwon, Sangmun Hwang, Dong Pyo Jang

**Affiliations:** 1 Department of Electronic Engineering, Hanyang University, Seoul, Republic of Korea; 2 Department of Biomedical Engineering, Hanyang University, Seoul, Republic of Korea; 3 Department of Healthcare Digital Engineering, Hanyang University, Seoul, Republic of Korea

**Keywords:** Neurotransmitter, Multichannel, Simultaneous measurement, Multiplexer, System development

## Abstract

Simultaneous measurement of neurotransmitters in multiple individuals enables more accurate comparison of behavioral and pharmacological responses between subjects. In this study, we developed a system capable of multichannel electrochemical measurements using multiplexers (MUXs). Although MUXs are widely used in analog circuits, their parasitic characteristics can degrade the accuracy of current-based electrochemical systems. Therefore, the effect of parasitic components on electrochemical measurements was examined through dopamine detection experiments and simulations using fast-scan cyclic voltammetry (FSCV). The experimental and simulation results showed that parasitic capacitance increased the background current and noise. In addition, a substantial background current increase of more than 5% was observed when the capacitance exceeded 50 pF. Based on the simulation results, MUX devices such as ADG1206 and TMUX6119, which have parasitic capacitance below 50 pF, were selected, and an optimized system was developed and evaluated. Verification experiments showed that the background current and noise of the optimized system were not significantly different from those of the standalone transimpedance amplifier (TIA) system. High channel uniformity and long-term stability were also achieved, with normalized root mean square error below 0.5%, normalized deviation within ± 3%, and Z-scores within ± 1. In dopamine detection experiments, rapid and consistent current responses verified the system’s reliable detection performance. With a 16-channel MUX configuration, the system supports multichannel simultaneous measurements using user-defined waveforms, including FSCV. It enables up to 11 channels at 10 Hz FSCV and up to 16 channels at 7 Hz FSCV. The proposed hardware can be integrated into existing systems, enabling scalable multichannel capability without the need for extensive redesign. Based on our experimental results, we demonstrated that the developed multichannel system provides reliable and effective detection. This study provides a foundational guideline for MUX-based electrochemical measurement systems. The proposed approach can support efficient experimental design in fields such as behavioral neuroscience and pharmacology by enabling simultaneous multichannel measurements.

**Supplementary Information:**

The online version contains supplementary material available at 10.1007/s13534-026-00554-3.

## Introduction

Electrochemical neurotransmitter measurement technology has been established as an essential tool in neuroscience and pharmacological research. In particular, fast-scan cyclic voltammetry (FSCV) has been increasingly used in vivo to monitor neurochemical changes associated with social interactions, stress, and drug treatments [1–5]. Barath et al. (2020) observed a rapid increase in phasic dopamine levels under hypoxic conditions induced during euthanasia using FSCV. Lichtenberg et al. (2018) applied the Pavlovian Social Distress paradigm to investigate dopamine changes in the nucleus accumbens core. Their study showed that in the presence of conspecifics, dopamine responses to cues predicting aversive stimulation decreased, whereas dopamine release increased when the conspecifics received the stimulation. These studies highlight the importance of analyzing neurotransmitter dynamics under social and stress-related conditions.

Maintaining consistent experimental conditions across multiple subjects is crucial for obtaining reliable results in behavioral and pharmacological studies. As such studies continue to expand, interest has grown in multichannel electrochemical measurement systems capable of simultaneously monitoring neurotransmitter changes in multiple subjects [6, 7]. However, most existing electrochemical neurotransmitter measurement systems are limited to single-subject recordings or a small number of channels [8–11]. While these systems are effective for small-scale experiments, their utility is restricted in studies requiring detailed measurements across a larger number of subjects. For multichannel systems to be effectively applied in multi-subject experimental paradigms, they must support simultaneous measurements, minimize signal interference, and maintain waveform fidelity. Such platforms are essential for identifying variability and consistency in neurotransmitter responses across diverse behavioral and physiological conditions, and they also improve experimental efficiency, reproducibility, and statistical robustness in cross-subject analyses [12, 13]. Consequently, there is a growing interest in scalable and application-specific electrochemical systems capable of synchronized measurements across multiple subjects.

Multiplexers (MUXs) are commonly used components in electronic circuits that can selectively route multiple input signals. This functionality simplifies circuit design and helps reduce hardware requirements [14]. Based on structural advantages, MUXs can be used in electrochemical measurement systems to sequentially process signals from multiple electrodes via a single amplifier and an acquisition circuit. By reducing the number of physical channels and the overall system complexity, MUX-based systems simplify system design. They also enable simultaneous multichannel measurements without requiring additional acquisition circuitry or external equipment.

However, MUXs are originally designed for digital signals or constant-voltage circuits, and therefore face several technical challenges when applied in electrochemical measurement environments. In particular, parasitic components, such as on-resistance and drain-source capacitances, can introduce RC delays between the electrode and the amplifier [15, 16]. RC delay increases the rising and falling times of the applied waveform and generates displacement current proportional to the rate of voltage change. As a result, waveform distortion, increased current noise, and delayed signal responses may occur. Although these issues may be negligible in typical voltage-based circuits, they can significantly reduce measurement sensitivity and repeatability in electrochemical systems that require quantitative current measurements in the nA to µA range. Therefore, selecting MUX components with appropriate resistance and parasitic capacitance is essential for implementing a precise, distortion-free electrochemical measurement system.

In this study, we developed a multichannel electrochemical measurement system optimized for neurotransmitter detection by selecting MUX components suitable for electrochemical environments. The proposed system induces adsorption of neurotransmitters via a holding potential. It sequentially delivers FSCV waveforms to multiple electrodes through MUX switching, enabling synchronized multichannel acquisition using a single data acquisition (DAQ) device. In addition, equivalent circuit simulations were performed to evaluate the effects of internal MUX electrical properties on electrochemical signals. Through these simulations, the current increases and voltage delays caused by parasitic capacitance and on-resistance during FSCV measurements were quantitatively analyzed. Based on the simulation results, optimized MUX components with improved signal fidelity compared to general-purpose devices were selected and implemented in a multichannel measurement platform.

## Method

### Data acquisition system

Data acquisition was performed using a National Instruments NI USB-6363 DAQ device (Austin, TX, USA), which provides up to 2 MS/s analog input and 2.86 MS/s analog output performance. Custom software developed in LabVIEW 2016 (National Instruments) was used to generate voltage waveforms, control the MUX system, and acquire current signals. The waveform output was delivered to up to 16 channels via the MUX, and the resulting current signals were converted to voltage using a transimpedance amplifier (TIA). These signals were digitized and displayed in real time as current values.

### Simulation

To investigate how parasitic capacitance and resistance in the MUX affect signal quality, we performed simulations based on an equivalent circuit model comprising the electrode, MUX, and TIA. LTspice XVII (Analog Devices, Inc., Norwood, MA, USA) was used to evaluate how variations in MUX parasitic components influence waveform propagation and measured current. The MUX was modeled using a first-order RC model [17], with the on-resistance and input capacitance values taken directly from the device datasheets. This simplified RC approach allowed us to isolate the contribution of parasitic elements while preserving the full electrode–TIA pathway. The electrical characteristics of the 7 μm carbon fiber microelectrodes (CFMEs) were obtained from electrochemical impedance spectroscopy (EIS) measurements. The impedance spectra were fitted to extract the solution resistance, charge transfer resistance and double-layer capacitance, which were then incorporated into the equivalent circuit model.

The simulations tested capacitance values of 0, 5, 10, 50, 100, and 200 pF, and resistance values of 0, 5, and 200 Ω. To closely replicate actual measurement conditions, the input waveform was defined as a − 0.4 to 1.3 V FSCV signal with a scan rate of 400 V/s.

### System validation

To validate the performance of the developed system, signal acquisition was conducted using both dummy cells and conventional beaker-based setups. For system validation, we employed the standard FSCV waveform commonly used in neurochemical measurements. The standard FSCV waveform is a triangular scan sweeping from − 0.4 V to 1.3 V and back at a scan rate of 400 V/s [18–20]. While up to 11 channels can typically be measured simultaneously at a conventional 10 Hz scan rate, this study reduced the scan rate to 7 Hz to enable validation across all 16 channels. The waveform was generated at 100 ksps, and the corresponding current was recorded, resulting in 851 current samples per scan.

To evaluate background current and noise characteristics, FSCV waveforms were applied in a beaker setup using a 7 μm CFME as the working electrode and an Ag/AgCl reference electrode. The background current was defined as the peak current value near 0.1 V. Signal noise was quantified as the standard deviation (SD) of the time-varying current at 0.6 V, which corresponds to the typical dopamine oxidation potential. For each electrode, noise was calculated from a 5-minute recording. The current was divided into five 1-minute segments, the SD was computed for each segment, and these five values were averaged to obtain the final noise level. 

 Dummy cells composed of identical resistive and capacitive components were used to assess both inter-channel uniformity and long-term signal stability. To evaluate channel uniformity, signals from all 16 channels were recorded simultaneously for approximately seven minutes, and the first five minutes of data (2100 scans) were used for analysis.

The normalized root mean square error (NRMSE) was computed for each channel using Eq. (1) to quantify waveform similarity across channels. Channels with NRMSE values below 0.5% were considered to exhibit acceptable uniformity [21]. To ensure robustness against potential noise or outliers, the normalization denominator was defined using an interquartile range (IQR) based range derived from the reference trace $\:\stackrel{-}{X}$. $\:\stackrel{-}{X}$ is the element-wise average voltammogram trace across all channels, computed for each time sample. $\:{X}_{IQR}$ is defines as the subset of data points in $\:\stackrel{-}{X}$ that fall within the IQR-based range $\:[{Q}_{1}-1.5IQR\le\:x\le\:\:{Q}_{3}+1.5IQR]$. The denominator was set to the difference between the minimum and maximum values within this robust $\:{X}_{IQR}$ range.

1 $$\:NRMSE=\frac{\sqrt{\frac{1}{N}{\sum\:}_{i=1}^{N}{\left({X}_{k,i}-{\stackrel{-}{X}}_{i}\right)}^{2}}}{\mathrm{max}\left({X}_{IQR}\right)-\mathrm{min}\left({X}_{IQR}\right)}\times\:100\:\left[\%\right]$$

where $\:{X}_{k}$ is the average voltammogram trace (a vector with N data points) of the k-th channel averaged over 2100 scans.

To further examine the temporal consistency of the signals and identify any potential outliers at specific voltage points, we calculated the normalized deviation between each channel’s response and the overall mean, as defined in Eq. (2).

2 $$\:{U}_{k,\:\:i}=\frac{{X}_{k,i}-\:{\stackrel{-}{X}}_{i}}{\mathrm{max}\left({X}_{IQR}\right)-\mathrm{min}\left({X}_{IQR}\right)}\times\:100$$

where $\:{U}_{k,i}$ is the normalized deviation of the k-th channel at sample * i*. $\:{X}_{k}$ is the average voltammogram trace of the k-th channel averaged over 2100 scans and $\:\stackrel{-}{X}$ is the element-wise average voltammogram trace used to define the normalization range $\:{X}_{IQR}$.

Long-term measurement stability was assessed using current values at 0.6 V extracted from continuous 100-hour recordings. Z-scores were computed by using the first 10-minute current as the baseline segment, and the baseline mean ($\:{\mu\:}_{0}$) and standard deviation ($\:{\sigma\:}_{0}$) were computed. For every subsequent 5-hour period, the mean current value $\:{x}_{t}$ was calculated from a 10-minute recording segment, and a Z-score was determined using the following expression.

3 $$\:{Z}_{t}=\frac{{x}_{t}-{\mu\:}_{0}}{{\sigma\:}_{0}}$$

To assess the consistency of signal acquisition across channels in a realistic experimental setup, we performed sequential measurements using a single electrode, connecting it to each channel one at a time. The dopamine detection experiment was performed using a beaker-based setup. The FSCV waveform was continuously applied to allow the current response to reach a stable baseline before dopamine was added. After the baseline stabilized, 1 µM dopamine was injected into the Tris buffer, and the resulting oxidation current was measured. To exclude transient fluctuations immediately following dopamine injection, the faradaic current was analyzed starting approximately 300 scans after injection. The dopamine oxidation current was quantified as the peak current near 0.6 V in the background-subtracted voltammogram.

To evaluate simultaneous measurement performance, dopamine signals were recorded in parallel using multiple electrodes. Due to experimental limitations, measurements were performed in two separate runs, each consisting of eight electrodes uniformly arranged within the beaker. After 1 µM dopamine was added, the current increase was monitored across all eight channels. The temporal difference was evaluated by comparing the onset of the current rise between the earliest- and latest-responding channels.

### Reagents and sample preparation

Dopamine solutions were prepared using a Tris buffer maintained at pH 7.4. The buffer consisted of 15 mM Trizma phosphate, 3.25 mM KCl, 140 mM NaCl, 1.2 mM CaCl₂, 1.25 mM NaH₂PO₄, 1.2 mM MgCl₂, and 2.0 mM Na₂SO₄. All reagents were obtained from Sigma-Aldrich (St. Louis, MO, USA). Dopamine stock solutions were prepared at 1 mM and diluted to the desired concentrations for each experiment. To prevent dopamine oxidation and improve storage stability, 50 µM perchloric acid was added to the solution.

### Carbon fiber microelectrode electrode fabrication

CFMEs were fabricated following previously established protocols [22]. AS4 carbon fibers (Hexcel, Stamford, CT) with a diameter of 7 μm were used. Approximately 100 μm of the fiber was exposed and trimmed using a surgical scalpel. Before use, the electrodes underwent electrochemical activation via surface functionalization by applying a FSCV waveform that swept from − 0.4 V to 1.5 V at a scan rate of 400 V/s and a frequency of 30 Hz. A secondary stabilization step was performed by applying the FSCV waveform from − 0.4 V to 1.3 V at a scan rate of 400 V/s at a frequency of 10 Hz.

### Signal processing and statistical analysis

All signal processing was performed using MATLAB (MathWorks Inc., Natick, MA), while statistical analyses were conducted with GraphPad Prism 10 (GraphPad Software, San Diego, CA). Data are presented as mean ± SD, with the number of replicates (n) indicated in the main text and figure captions.

Prior to hypothesis testing, the assumptions of normality and homogeneity of variance were evaluated using the Shapiro–Wilk test and F-test, respectively. Parametric tests were applied only when the normality assumption was met, whereas nonparametric tests were used when it was not. Homogeneity of variance was used to determine whether Welch’s t-test or a standard paired-comparison test was appropriate.

To quantify changes in background current in the MUX system, current values were normalized to the background current measured at each electrode in the standalone TIA system. The normalized value for the TIA system was set to 1 as a reference. Accordingly, comparisons involving the standalone TIA system were evaluated using one-sample t-tests. Whereas direct comparisons between the pre-optimized and optimized MUX configurations were assessed using the Student’s t-test after confirming normality and equal variances.

For noise analysis, datasets from the standalone TIA system, pre-optimized MUX system, and optimized MUX system were first assessed using the Shapiro–Wilk test to confirm normality. Because the sample sizes differed across systems, noise levels were compared using the Brown–Forsythe and Welch’s ANOVA, followed by Dunnett’s T3 multiple comparisons test to account for unequal variances.

### Power and printed circuit board

The system was powered from a + 15 V source. The initial design generated both ± 5 V and ± 3 V rails on the board. An ADP5073 inverting converter generated − 5 V. A 2.2 µH inductor (XAL5050-223MEC), a DFLS240-7 Schottky diode, and a 10 µF ceramic capacitor filtered the output. Two LP2986 regulators produced + 5 V and + 3 V. A LM2776 charge-pump inverter generated − 3 V. The ADG1419 MUX was supplied with ± 5 V, and the ADG706 MUX was supplied with ± 3 V, ensuring operation within the fully specified supply-voltage ranges for both devices. In the optimized system, a ± 15 V power module based on the TPS5430 converter replaced the internal generation stage. This module provided stable supply rails with lower ripple.

The MUX system and printed circuit board (PCB) were designed using Altium Designer (Altium LLC, San Diego, CA, USA). To reduce analog signal noise, the PCB was configured as a four-layer board, with analog signal traces on the top layer, ground on the second layer, power on the third layer, and digital signals on the bottom layer. Signal ground and power ground were isolated from one another to minimize interference. All switching rails were also filtered and routed separately from the signal paths to reduce coupling.

### Equivalent capacitance model for the two-stage multiplexer configuration

In the experimental system, two analog MUXs were connected in series, each contributing an intrinsic on-capacitance ($\:{C}_{MUX1}$ and $\:{C}_{MUX2}$) at its internal switching node. For comparison with the single-capacitance simulation model, the total capacitive loading introduced by the signal passes through two MUXs in series was expressed as an equivalent capacitance ($\:{C}_{eq}$). Although the capacitors reside at different nodes within an R–C ladder, the effective capacitive load experienced by the input waveform can be approximated by the parallel sum $\:{C}_{eq}={C}_{MUX1}+{C}_{MUX2}$.

This approximation is supported by the relative timescales of the system. The characteristic RC time constants (*τ*) of the MUX branches are on the order of a few nanoseconds, corresponding to cutoff frequencies well above tens to hundreds of megahertz. In contrast, common electrochemical waveform techniques operate at much lower frequencies. Methods such as square wave voltammetry, potential step experiments, and fast voltammetry remain well below the kilohertz range. Because the excitation frequencies satisfy $f\:\ll\:1/2\pi\tau\:$, the internal node voltages in the cascaded MUX chain follow the input waveform with negligible attenuation. Under these quasi-static operating conditions, both capacitors experience nearly identical voltage excursions, and the resulting displacement current is given by Eq. (4).

4 $$\:i\left(t\right)\approx\:\left({C}_{1}+{C}_{2}\right)\frac{dV}{dt}$$

    

Thus, the two-stage MUX chain imposes an effective capacitive load that is accurately represented by the sum $\:{C}_{eq}={C}_{MUX1}+{C}_{MUX2}$, which was used to compare the experimental background-current scaling with the single-capacitor simulation model.

## Results

### MUX system architecture

**Fig. 1:**
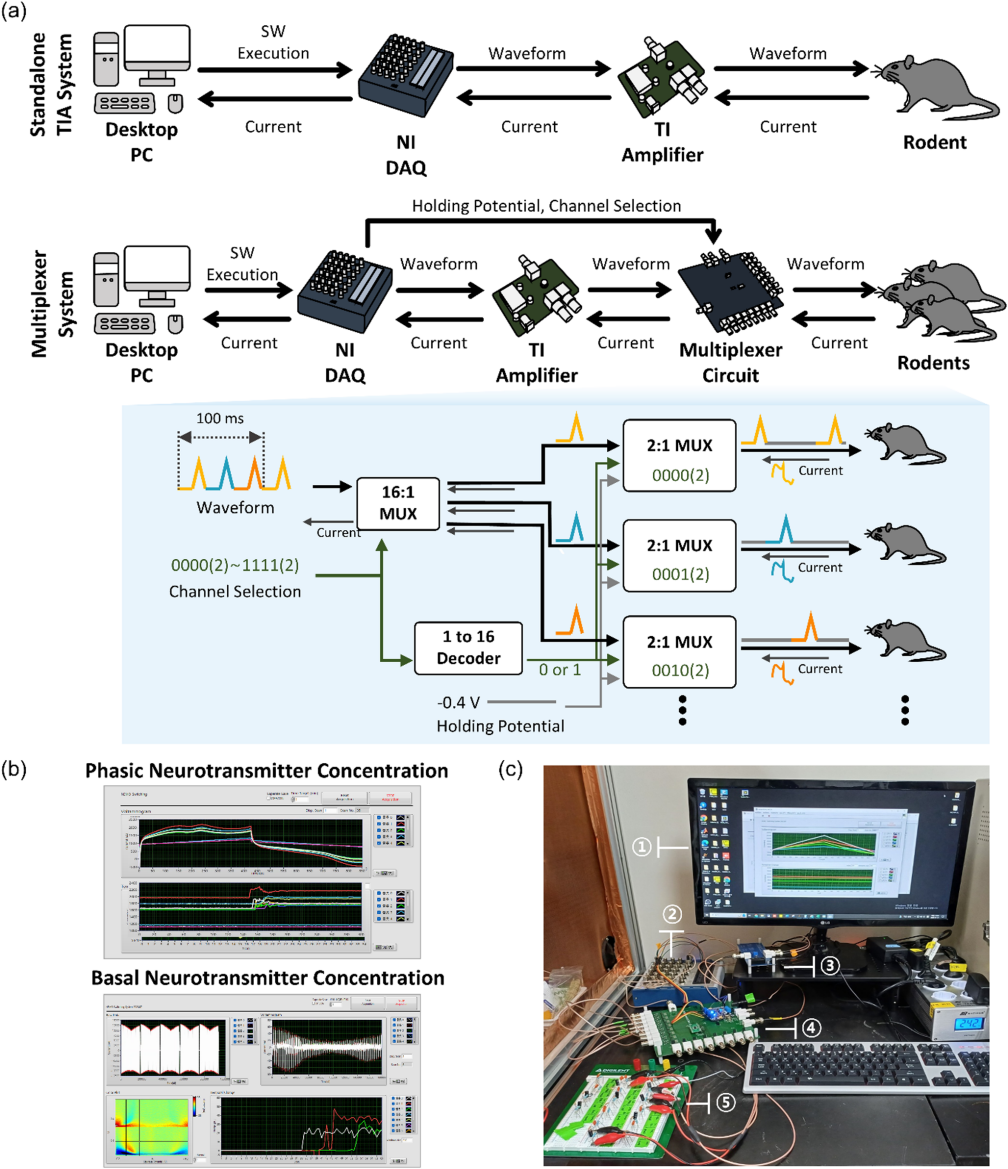
Configuration of the MUX-based multichannel simultaneous measurement system; **a** Circuit diagram comparing the conventional TIA-only system for single-subject FSCV measurement with the proposed multichannel system. While the conventional setup supports only single-subject acquisition, the proposed design enables sequential multichannel measurement by integrating MUX circuits with a single DAQ device, **b** LabVIEW 2016–based user interface providing integrated control of waveform selection, DAQ and MUX operation, and real-time signal visualization, **c** Photograph of the system operating with dummy cells. The system comprises ① the UI, ② DAQ device, ③ TIA circuit, ④ MUX module, and ⑤ connected dummy cells

To address the limitations of existing single-subject measurement systems, this study developed a multichannel electrochemical measurement system capable of simultaneously recording neurotransmitter activity from multiple animals. As shown in Fig. 1(a), in the conventional configuration, the TIA is directly connected to the DAQ device, which restricts measurements to a single subject. In contrast, the system developed in this study enables a single DAQ device to sequentially record signals from multiple animals by integrating a MUX module after the TIA.

The MUX configuration includes a 16:1 MUX paired with sixteen 2:1 MUXs. The DAQ generates two waveforms, a measurement waveform and a holding potential. The measurement waveform is routed through the TIA to the 16:1 MUX, which selects one of the 16 channels. The electrode on the selected channel receives the measurement waveform through its corresponding 2:1 MUX. For all non-selected channels, the 2:1 MUX switches to the holding-potential path, ensuring each electrode maintains continuous neurotransmitter adsorption.

Custom LabVIEW software was developed to enable sequential measurements from multiple subjects within a single waveform cycle. With an FSCV waveform operating at 10 Hz, resulting in a 100 ms cycle time, a single scan (from − 0.4 V to 1.3 V, 400 V/s) requires approximately 8.5 ms, calculated from the total voltage excursion (2 × (1.3 − (− 0.4)) / 400 = 8.5 ms). The remaining 91.5 ms of the cycle is available for the holding potential. During this interval, up to 10 additional waveforms can be applied (91.5 ms/8.5 ms ≈ 10.8), allowing measurements from 11 subjects per cycle (1 primary scan + 10 additional scan).

Fig. 1(b) shows the LabVIEW 2016 software interface, which integrates waveform selection, waveform generation, DAQ control, and digital switching of the MUX. Acquired data are visualized in real-time, with functionalities for channel selection, zooming, and overlay comparison.

As shown in Fig. 1(c), the fully assembled system is composed of the software interface, DAQ device, TIA circuit, MUX module, and dummy cell, and supports simultaneous measurement across up to 11 channels at 10 Hz using an FSCV waveform (− 0.4–1.3 V, 400 V/s).

### Electrochemical evaluation of general-purpose MUX components

During the design phase, we prioritized key parameters, including low on-resistance, minimal crosstalk, and high switching speed. Based on these criteria, we selected the ADG706 and ADG1419 (Table 1), which are commonly used in analog switching circuits due to their low on-resistance and fast performance. To evaluate their suitability for electrochemical measurements, we constructed a pre-optimized MUX system using these components and assessed its performance with respect to background current and noise.

**Table 1:** Key electrical specifications of general-purpose multiplexer components ADG706 and ADG1419

	ADG 706	ADG 1419
Channel count	16:1	2:1
Analog signal range	−3 V to + 3 V	−5 V to + 5 V
Transition Time ($\:{\mathrm{t}}_{\mathrm{T}\mathrm{R}\mathrm{A}\mathrm{N}\mathrm{S}\mathrm{T}\mathrm{I}\mathrm{O}\mathrm{N}}$) ($\:{R}_{L}=300\:{\Omega\:},\:{\mathrm{C}}_{\mathrm{L}}=35\:pF$)	40 ns	310 ns
On Resistance ($\:{\mathrm{R}}_{\mathrm{O}\mathrm{N}}$)	2.5 $\:{\Omega\:}$	4.5 $\:{\Omega\:}$
Drain, Source Capacitance ($\:{\mathrm{C}}_{\mathrm{D}}$, $\:{\mathrm{C}}_{\mathrm{S}}$)	200 pF	128 pF
Drain Off Leakage Current ($\:{\mathrm{I}}_{\mathrm{D}\left(\mathrm{O}\mathrm{F}\mathrm{F}\right)}$)	± 0.01 nA	± 0.1 nA
Channel-to-Channel Crosstalk	−80 dB	−60 dB
-3 dB Bandwidth ($\:{R}_{L}=50\:{\Omega\:},\:{\mathrm{C}}_{\mathrm{L}}=5\:pF$)	25 MHz	105 MHz

In these tests, the CFME was immersed in a Tris buffer solution, and an FSCV waveform (− 0.4 to 1.3 V, 400 V/s) was applied to measure the background current and signal noise. The background current was defined as the peak current near 0.1 V. The entire voltammogram was then normalized by dividing all current values by the peak background current measured in the standalone TIA system. Therefore, the peak near 0.1 V in the standalone TIA system becomes 1 after normalization.

**Fig. 2:**
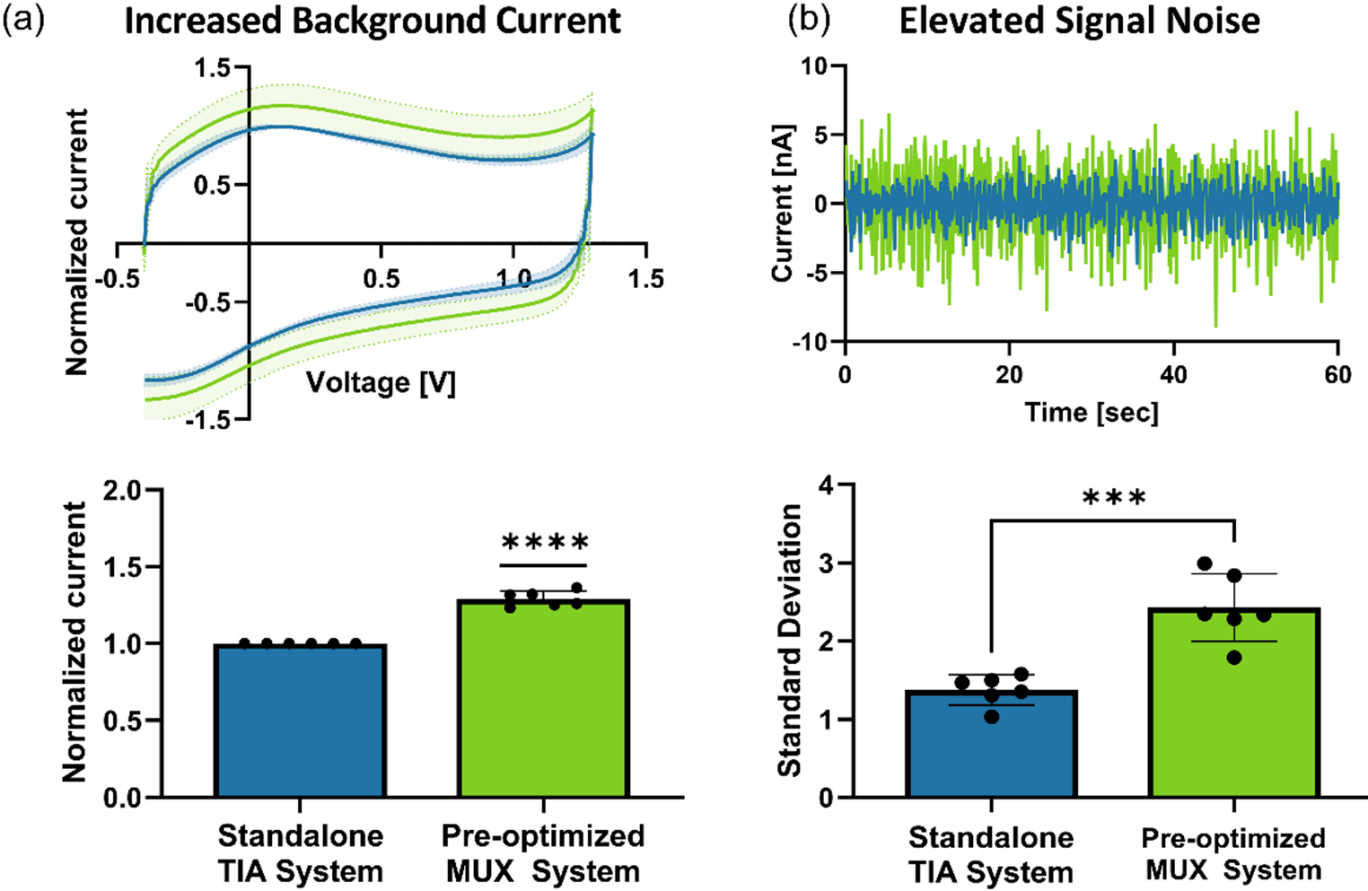
Comparison of background current and noise between the TIA-only and unoptimized MUX systems; Normalized to the TIA-only configuration, the unoptimized MUX system exhibited a 1.29-fold increase in background current and a 1.77-fold increase in noise (*n* = 6), attributable to elevated parasitic capacitance and additional interference pathways introduced by the MUX components. ****p < 0.0001 (one-sample t-test); ***p < 0.001 (Student’s t-test)

Compared to the standalone TIA system, the pre-optimized MUX system exhibited a 1.29-fold increase in background current (1.29 ± 0.05; *n* = 6; one-sample t-test, *p* < 0.0001). In terms of noise, the standalone TIA system showed a level of 1.37 ± 0.19 nA, whereas the pre-optimized MUX system showed 2.43 ± 0.43 nA, corresponding to a 1.77-fold increase (*n* = 6; Student’s t-test, *p* = 0.0003) (Fig. 2).

These increases are attributed to displacement current induced by parasitic capacitance in the MUX. The elevated noise likely stems from both amplified baseline currents and additional interference paths introduced by the MUX.

Such signal degradation poses challenges for high-precision in vivo measurements, underscoring the need for selection criteria tailored specifically to electrochemical systems beyond conventional electronic specifications.

### Equivalent circuit-based simulation

**Fig. 3:**
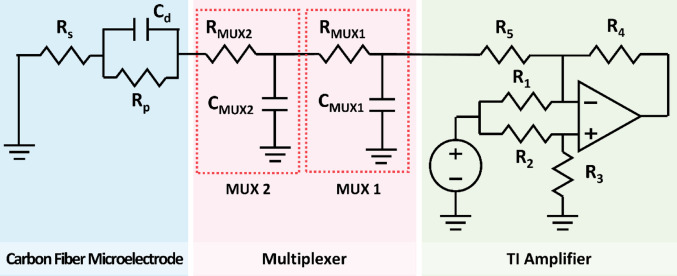
Equivalent circuit model incorporating MUX and electrode characteristics; Simulation-based equivalent circuit model developed to quantitatively assess the impact of MUX insertion on electrochemical signal integrity. The model includes the impedance characteristics of a 7 µm CFME, the parasitic resistance and capacitance of the 16:1 and 2:1 MUX components, and the TIA circuit

To quantify the influence of MUX parasitic characteristics and guide system design, we conducted simulations using LTspice XVII (Analog Devices, Inc., Norwood, MA). As in actual experiments, the input signal was configured as a − 0.4 to 1.3 V FSCV waveform at a scan rate of 400 V/s. The equivalent circuit model comprises a 7 μm CFME impedance model, a TIA circuit, and 16:1 and 2:1 MUX elements, all of which are modeled with adjustable parasitic components (Fig. 3). The equivalent model incorporated the CFME impedance obtained from EIS measurements, including the fitted solution resistance, charge transfer resistance and double-layer capacitance. This simplified RC representation isolates the incremental distortion introduced by the MUX while retaining the essential electrode–TIA signal pathway. It allows a direct assessment of how parasitic capacitance affects waveform fidelity and the resulting current responses.

**Fig. 4:**
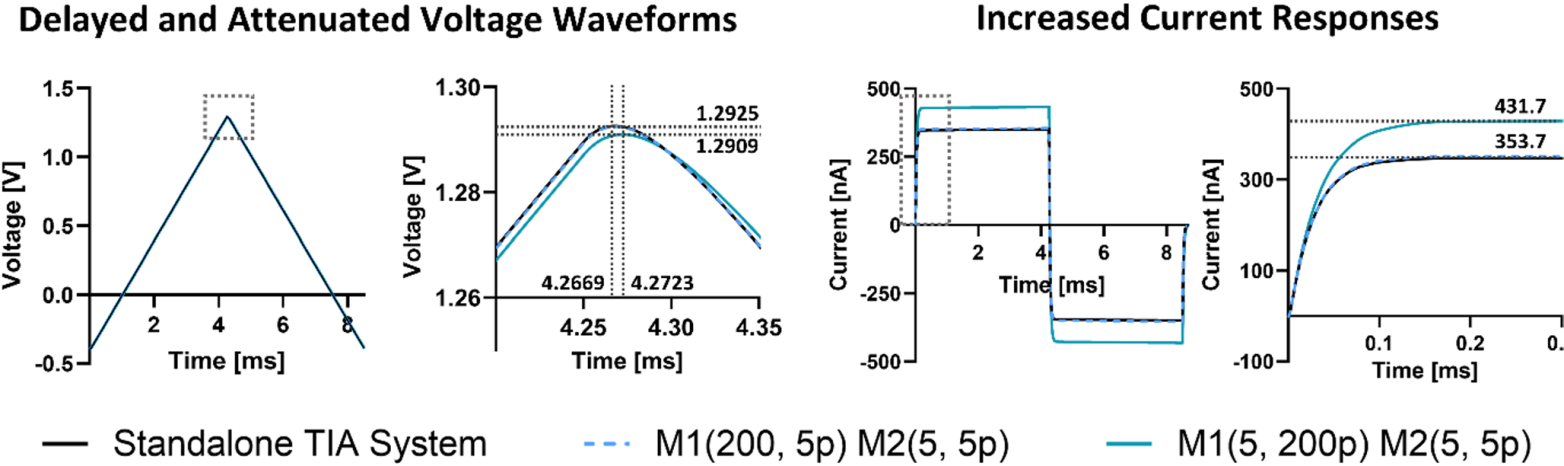
Effect of MUX on-resistance and parasitic capacitance on voltage and current responses; Variations in the on-resistance of MUX 1 produced negligible changes in both the applied voltage and resulting current. In contrast, increasing the parasitic capacitance to 200 pF caused slight distortion in the applied voltage waveform and a corresponding increase in the measured current

Before conducting the parameter sweep shown in Fig. 4, we verified that the relative ordering of the two multiplexers did not affect the resulting waveform distortion. As shown in Figure S1, swapping the high-RC and low-RC MUX elements yielded identical simulation results, confirming that the two-stage configuration is structurally symmetric. Therefore, in subsequent simulations, MUX 2 was fixed at its minimum values (5 Ω, 5 pF), and only MUX 1 parameters were varied.

Fig. 4 presents the simulation outcomes for different resistance and capacitance values. MUX 1 parameters were varied from 0 to 200 Ω and from 0 to 200 pF, while MUX 2 was fixed at 5 Ω and 5 pF. Increased resistance had a negligible impact on signal shape, whereas increasing capacitance caused waveform distortion and elevated current levels. Specifically, the peak voltage decreased by 0.12%, and the response delay increased by 0.13%. In contrast, the current increased by 22.05%, confirming that parasitic capacitance is the dominant contributor to distortion.

**Fig. 5:**
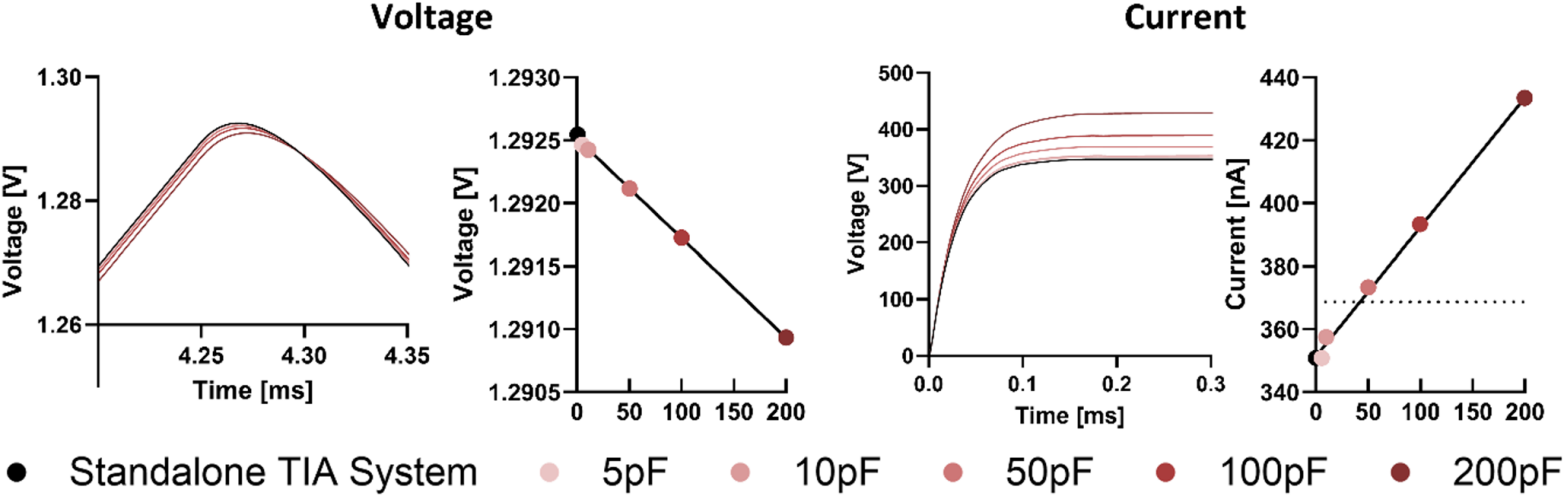
Analysis of voltage and current distortion trends as a function of parasitic capacitance; Linear regression lines were included to illustrate the observed trends. As parasitic capacitance increased, peak voltage decreased linearly while current amplitude increased linearly. Although the voltage reduction remained within 1%, the current exceeded a 5% increase relative to the standalone TIA system when capacitance surpassed 50 pF

After identifying capacitance as the primary factor, the model was simplified to a single-MUX configuration to quantify distortion more generally (Fig. 5). Additional simulations were performed with capacitance values of 5, 10, 50, 100, and 200 pF, spanning the range of commercially available MUX components. As the result, voltage deviations remained below 1% across all conditions, whereas current distortion exceeded 5% for capacitances above 50 pF. Based on the 95% confidence interval, a current change within 5% was defined as the allowable range. Using this criterion, the optimized system was designed such that the effective capacitance of the entire MUX configuration remained below 50 pF.

### Optimized electrochemical MUX system

The simulation results (Fig. 5) indicate that parasitic capacitance is the dominant factor affecting current distortion. Accordingly, we selected two optimized MUX components with low parasitic capacitance and high channel isolation, ADG1206 (15 pF, Analog Devices) and TMUX6119 (8.1 pF, Texas Instruments) (Table 2).

**Table 2:** Specification comparison of optimized multiplexer components ADG1206 and TMUX6119 for electrochemical systems

	ADG 1206	TMUX 6119
Channel Count	16:1	2:1
Analog Signal Range	−15 V to + 15 V	−15 V to + 15 V
Transition Time ($\:{\mathrm{t}}_{\mathrm{T}\mathrm{R}\mathrm{A}\mathrm{N}\mathrm{S}\mathrm{T}\mathrm{I}\mathrm{O}\mathrm{N}}$) ($\:{R}_{L}=300\:{\Omega\:},\:{\mathrm{C}}_{\mathrm{L}}=35\:pF$)	80 ns	68 ns
On Resistance ($\:{\mathrm{R}}_{\mathrm{O}\mathrm{N}}$)	120 $\:{\Omega\:}$	140 $\:{\Omega\:}$
Drain, Source Capacitance ($\:{\mathrm{C}}_{\mathrm{D}}$, $\:{\mathrm{C}}_{\mathrm{S}}$)	13 pF	6.4 pF
Drain Off Leakage Current ($\:{\mathrm{I}}_{\mathrm{D}\left(\mathrm{O}\mathrm{F}\mathrm{F}\right)}$)	± 0.05 nA	± 0.005 nA
Channel-to-Channel Crosstalk	-85 dB	-93 dB
-3 dB Bandwidth ($\:{R}_{L}=50\:{\Omega\:},\:{\mathrm{C}}_{\mathrm{L}}=5\:pF$)	280 MHz	700 MHz

**Fig. 6:**
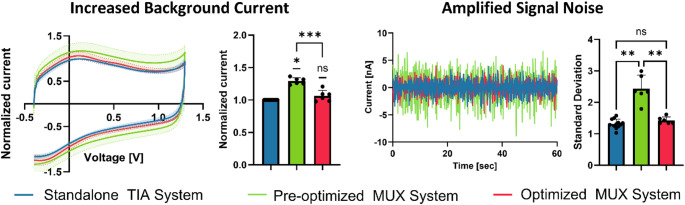
Comparison of background current and noise across the standalone TIA, pre-optimized MUX, and optimized MUX systems; The pre-optimized MUX system exhibited a significant increase in background current (*n* = 6), whereas the optimized system maintained levels comparable to the standalone TIA (*n* = 12). Noise amplitude was markedly reduced in the optimized system (*n* = 6), showing no statistical difference from the standalone TIA. ***p < 0.001 (Student’s t-test), *p < 0.1, ns > 0.05 (one-sample t-test); **p < 0.01, ns > 0.05 (Dunnett’s T3 multiple comparisons test)

To verify their electrochemical suitability, we analyzed background current and noise using the same protocol as in previous experiments. As shown in Fig. 6, background current was significantly lower in the optimized MUX system (*n* = 6; 1.06 ± 0.09) than in the pre-optimized system (*n* = 6; Student’s t-test; *p* = 0.0002), and not statistically different from the standalone TIA system (*n* = 12; one-sample t-test; *p* > 0.05). Likewise, noise levels were reduced in the optimized system compared to the pre-optimized version (1.42 ± 0.11 nA; Brown-Forsythe ANOVA test; *p* = 0.0004; Welch’s ANOVA test; *p* = 0.0005; Dunnett’s T3 multiple comparisons test; *p* = 0.004 for pre-optimized MUX system vs. optimized MUX system) and remained statistically equivalent to the TIA-only configuration (Brown-Forsythe ANOVA test; *p* = 0.0004; Welch’s ANOVA test; *p* = 0.0005; Dunnett’s T3 multiple comparisons test; *p* > 0.05 for standalone TIA system vs. optimized MUX system). These results confirm that the optimized MUX components maintain signal integrity.

**Fig. 7:**
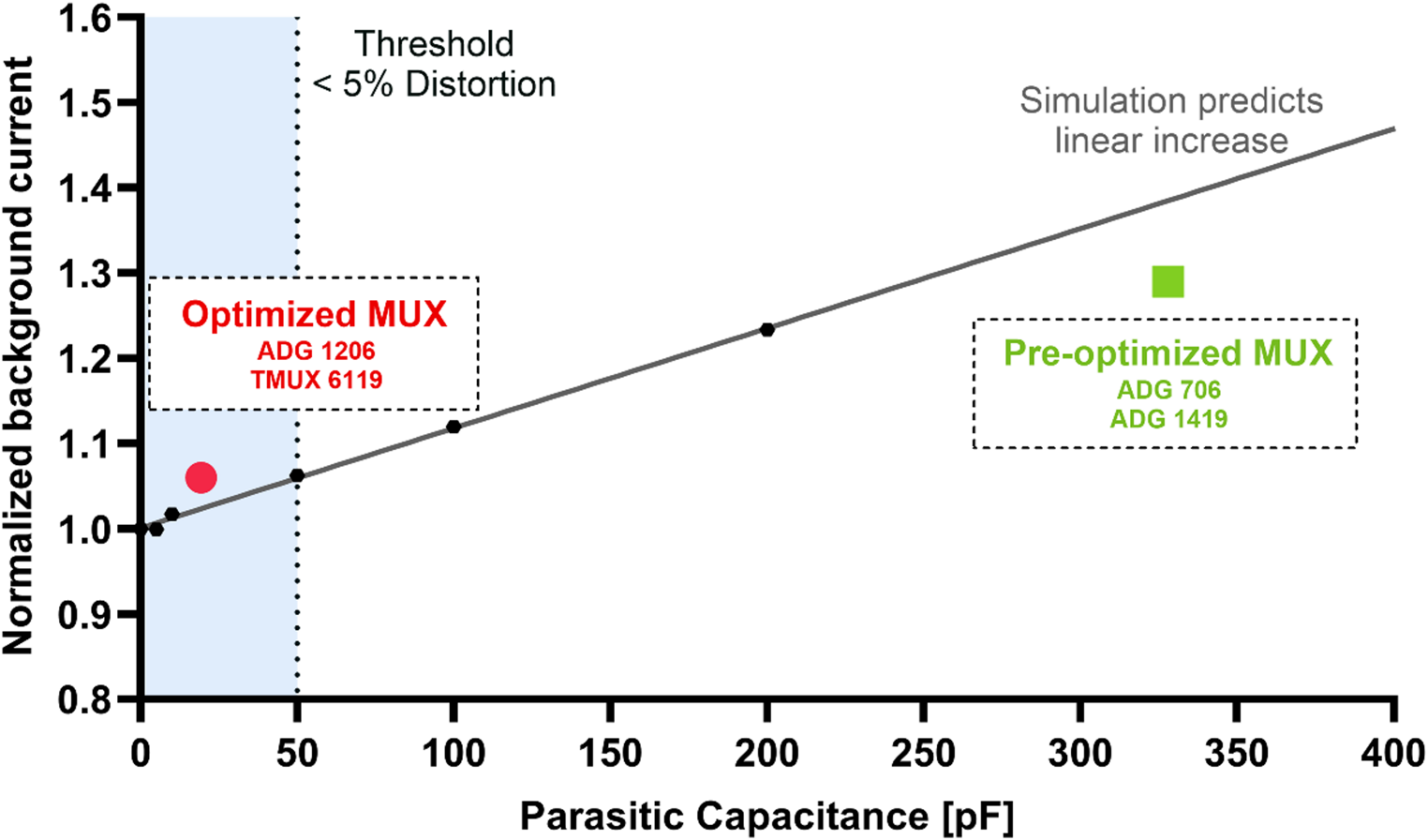
Summary of parasitic capacitance–dependent distortion obtained from simulation and experiment; Simulation results showed a linear increase in background current with increasing capacitance and identified 50 pF as the distortion threshold (> 5% deviation). Effective capacitance values were computed for each MUX configuration using the equivalent model: the pre-optimized MUX system (328 pF) exhibited a 29% increase in background current, whereas the optimized MUX system (19.4 pF) showed only a 6% increase. Although absolute magnitudes differed from the ideal RC model, the overall distortion trend was consistent with the simulation prediction

Fig. 7 summarizes the capacitance–current relationship predicted by the simulation and compares it with the experimentally measured background current changes. In the simulation, the background current increased linearly with parasitic capacitance, and capacitance values exceeding 50 pF produced increases of more than 5%, which was defined as the distortion threshold. The effective capacitance $\{C\}_-\{eq\}$ of each MUX configuration was calculated using the equivalent model described in the Methods section. The pre-optimized MUX system (ADG706, ADG1419) has input capacitances of 200 pF and 128 pF, respectively, for a combined value of 328 pF, which significantly exceeds the threshold. In contrast, the optimized MUX system had an effective capacitance of 19.4 pF (ADG1206: 13 pF; TMUX6119: 6.4 pF), well below the threshold. The experimentally measured background current changes exhibited a gentler slope than the simulated curve, but the overall trend remained consistent with the model. In the simulation, the pre-optimized MUX system in the high-distortion region showed a 29% increase in background current. On the other hand, the optimized MUX system, with an effective capacitance of 50 pF or less, showed a background current increase of 6%, within the allowable range. Since an electrochemical system does not consist solely of capacitive components but also includes factors such as electrode interfacial dynamics, the current increase observed in the experiments did not fully match the ideal RC model. Nevertheless, the distortion characteristics and relative performance of the two MUX configurations were consistent with the capacitance-dependent trends predicted in the simulation.

Additional simulations were performed to analyze further the frequency-dependent characteristics of the parasitic components (Figure S2). Frequencies of 1, 2, 3, 5, 10, and 50 Hz were evaluated to compare the effects of repetition frequency and parasitic capacitance. The simulation results showed that voltage and current variations were minimal, remaining below 0.5% across all capacitance groups. This result indicates that distortion was negligible because typical FSCV repetition frequencies are sufficiently lower than the MUX RC cutoff. In contrast, clear changes were observed in the simulations using scan rate as a variable (Figure S3). When evaluated at scan rates ranging from 100 to 5000 V/s, voltage attenuation increased linearly with scan rate and became more pronounced as capacitance increased. Meanwhile, the current amplitude followed the expected C·dV/dt relationship. After normalization, identical slopes were observed regardless of the magnitude of parasitic capacitance, indicating that parasitic capacitance primarily contributes to baseline shifts rather than altering the dynamic response. These additional simulations confirmed that parasitic capacitance and scan rate are the principal factors determining distortion in multichannel electrochemical systems.

### Experimental validation of the optimized MUX system

**Fig. 8:**
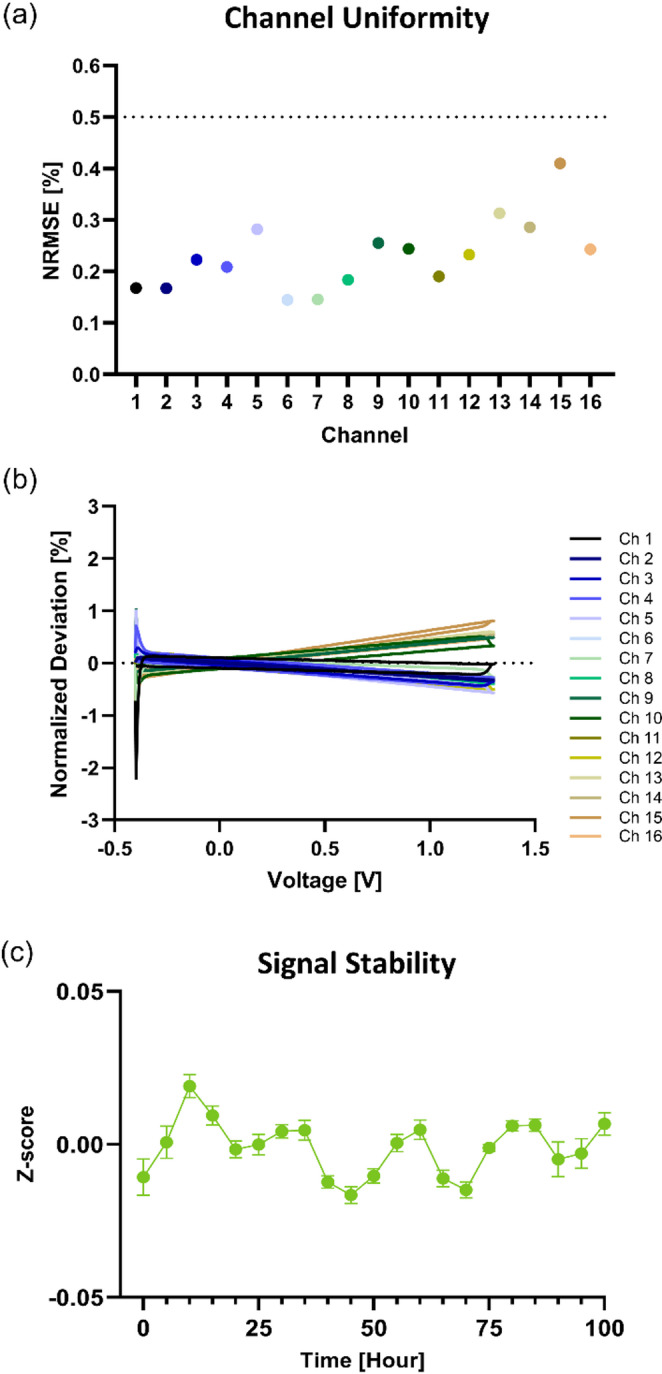
Evaluation of channel uniformity and long-term signal stability; **a**–**b** Sixteen channels were measured simultaneously for 10 min using identical dummy cells. **a** NRMSE and **b** normalized deviation were calculated. All channels exhibited NRMSE values below 0.5% and no deviations exceeding ± 3 were observed across the full waveform range, **c** The same dummy cells were measured continuously for 100 min, and Z-scores were calculated over time for each channel (mean ± SD). All values remained within ± 1, confirming stable long-term performance

To evaluate both inter-channel uniformity and long-term signal stability, we conducted measurements using 16 dummy cells fabricated with identical electrical characteristics. Fig. 8(a) and (b) present the NRMSE and normalized deviation. All channels exhibited NRMSE values below 0.5%, demonstrating strong alignment with the average waveform. The normalized deviation across voltage ranges remained within ± 3%, indicating minimal channel-to-channel variability in signal response.

For long-term stability assessment, continuous measurements were taken over 100 h using the same dummy cell setup. The current at each channel was converted to a Z-score using the baseline mean and standard deviation obtained from the first 10-minute segment. Z-scores were then calculated every 5 h from subsequent 10-minute data segments, as shown in Fig. 8(c). The values remained nearly constant across all channels, with differences staying within approximately 0.02. This demonstrates that the system maintains highly stable signal acquisition over extended durations. Taken together, the results in Fig. 7 validate the optimized MUX system’s reliability in terms of both signal consistency and temporal stability.

**Fig. 9:**
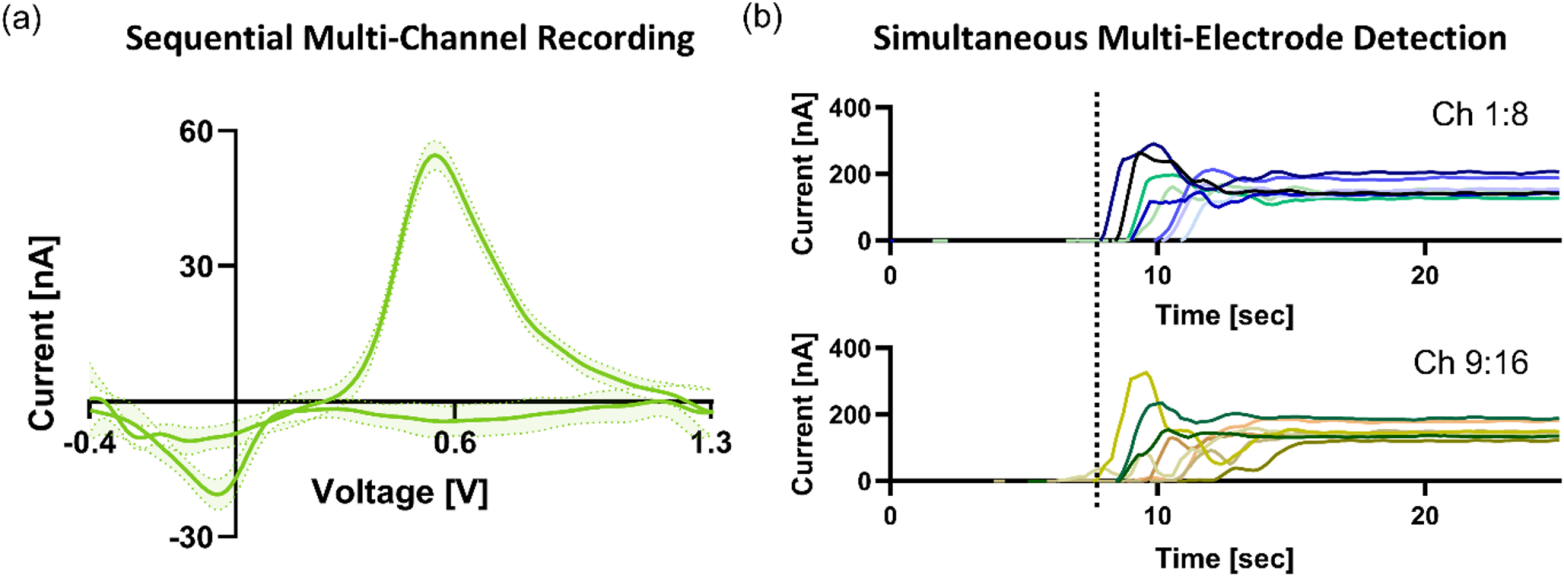
Multichannel and simultaneous dopamine measurement results; **a** Faradaic current responses obtained by sequentially connecting a single electrode to 16 channels for dopamine oxidation measurement. Data are presented as mean ± SD. At 0.6 V, the average current was 49.3 nA with a SD of approximately 3.2 nA. **b** Simultaneous measurement results were obtained by immersing 8 electrodes in a single beaker and injecting dopamine. The experiment was conducted in two rounds of 8 channels each. The onset of current increase differed by up to 5 s between channels

To further assess the system’s multichannel performance under electrochemical conditions, dopamine oxidation currents were measured in two complementary experiments. In Fig. 9(a), a single electrode was sequentially connected to all 16 channels under identical conditions. The average faradaic current at 0.6 V was approximately 49.3 nA, with a SD of 3.2 nA. While this variability slightly exceeded the predefined ± 3% threshold for system precision, it is likely due to minor instabilities from electrode reuse, rather than inherent system limitations.

In a separate experiment presented in Fig. 9(b), measurements were performed in two groups of eight channels due to experimental limitations. The eight electrodes were stabilized after being immersed in the same beaker, and following stabilization, 1 µM dopamine was injected into the center of the beaker for simultaneous detection. The current signals were aligned based on the onset of the signal rise. The difference in response time between the earliest and latest channels was approximately five seconds and showed a strong correlation with the distance of each electrode from the dopamine injection site. The earliest- and latest-responding electrodes were located at spatially opposite positions relative to the cluster of rapidly responding electrodes. This result suggests that physical factors such as the dopamine injection location, diffusion direction and speed, degree of solution mixing, and electrode arrangement influenced the response timing. Overall, most electrodes showed a current increase within 3 s of dopamine injection, indicating that the system provides sufficient temporal resolution and spatial sensitivity for real-time, simultaneous multichannel detection.

## Discussion

In this study, we developed a multichannel electrochemical measurement system capable of simultaneously detecting neurotransmitter signals from multiple subjects and proposed criteria for selecting and designing suitable MUX components. Our findings demonstrated that general-purpose MUX components commonly used in conventional electronics are likely unsuitable for electrochemical measurements.

Although the integration of MUX devices is essential for enabling multichannel acquisition, certain electronics characteristics prioritized in other applications can degrade performance in current-based electrochemical systems. Features such as low on-resistance and fast switching speed may introduce unintended distortions when applied to these measurements. In particular, the pre-optimized MUX components ADG706 and ADG1419, despite their favorable electrical specifications, resulted in significantly elevated background currents and increased noise levels, as shown in Fig. 2. These effects were attributed to RC charging/discharging behavior and displacement currents caused by parasitic capacitance.

Equivalent circuit simulations using LTspice (Figs. 3, 4 and 5) revealed that while changes in on-resistance had minimal impact on electrochemical signal quality, parasitic capacitance led to voltage waveform distortion and elevated measured currents. Notably, capacitance values exceeding 50 pF consistently led to current increases exceeding 5% relative to a standalone TIA system, well beyond typical statistical variation thresholds. Based on this observation, 50 pF was established as a practical threshold for component selection in electrochemical system design.

Following this analysis, we selected ADG1206 and TMUX6119 as optimized MUX components due to their low parasitic capacitance and high inter-channel isolation, as summarized in Table 2. Experimental validation showed that the optimized system’s background current and noise levels were statistically indistinguishable from those of the standalone TIA system, supporting the validity of the simulation-based design criteria (Fig. 6).

The capacitance–current relationship predicted by the simulation was directly compared with the experimental results (Fig. 7). Although the absolute current increase differed from the simulated values due to non-capacitive factors such as electrode interfacial kinetics, the capacitance-dependent behavior of each MUX configuration closely matched the simulation. The pre-optimized system, which significantly exceeded the 50 pF threshold, exhibited a large increase in background current, whereas the optimized system showed a change of only about 5%. These results confirm that the component selection criteria derived from circuit simulations can be effectively applied to practical system design.

To evaluate the performance of the final system, inter-channel uniformity and long-term measurement stability were assessed using identical dummy cells. Inter-channel uniformity was evaluated by assessing waveform consistency across all channels, and long-term stability was assessed by determining whether a constant value was maintained over an extended period. Across all channels, the NRMSE was less than 0.5%, and the normalized deviation was within ± 3%, indicating excellent inter-channel uniformity. In addition, the Z-score remained within ± 1 throughout the entire 100-hour dataset, demonstrating stable performance even during long-term measurements (Fig. 8).

The system’s performance was further verified through dopamine-detection experiments using actual electrodes. When a single electrode was sequentially connected to all 16 channels, the faradaic current at 0.6 V exhibited a standard deviation of approximately 3.2 nA. In a separate eight-channel simultaneous measurement, the difference in response time between the earliest and latest channels was within five seconds, confirming that the system provides sufficient temporal resolution (Fig. 9).

Previous studies on multichannel neurochemical sensing have primarily focused on electrode-array development. Zachek et al. (2010) demonstrated the ability to measure neurotransmitters at multiple spatial locations within a single subject using multichannel electrodes. Rafi and Zestos (2021) applied multiplexed waveforms to carbon-fiber multielectrode arrays to achieve region-specific sensing. FSCV systems capable of multi-analyte detection have also been reported, including decoupled dopamine–oxygen sensing using pyrolyzed carbon microarrays and dopamine–serotonin separation using glassy-carbon microelectrode arrays [23, 24]. More recently, hardware-level strategies for managing multiple electrochemical signals have been proposed. Avula et al. (2023) introduced a system that interleaves FSCV and electrophysiology through fast switching and optimized filtering, demonstrating the scalability of time-division approaches. However, most of these studies remain focused on single-subject measurements.

This study takes a different direction from prior work by enabling simultaneous measurements across multiple subjects. We developed a multichannel electrochemical measurement system based on commercially available MUX components that supports up to 16 channels and enables simultaneous monitoring of neurotransmitter concentrations in multiple subjects. This capability extends beyond traditional single-subject, multi-site recordings to true multi-subject acquisition, thereby improving statistical power and experimental efficiency in behavioral and pharmacological studies. In addition, the system allows user-defined waveforms to be applied without hardware modification, providing high flexibility for adapting to different analytes, stimulation paradigms, and experimental designs.

To ensure accurate electrochemical measurements, we evaluated how the electrical properties of several MUX devices affect waveform fidelity and background current. Although the influence of parasitic capacitance has been discussed in analog and bioimpedance circuits [25–27], its quantitative impact in electrochemical neurotransmitter measurements has been rarely analyzed. The simulations and experiments presented here provide practical insight for selecting MUX components in electrochemical systems. In particular, capacitance values exceeding 50 pF consistently produced measurable signal distortion, offering a useful engineering guideline for multichannel system implementation.

This study presents a multichannel electrochemical measurement system constructed entirely from commercially available components, offering scalable performance through stable signal acquisition and support for user-defined waveforms. These capabilities have not been demonstrated in previous array-based or CMOS-based approaches and offer a practical, reproducible foundation for future multichannel neurochemical experiments.

However, this study has several limitations. Only basic modeling of the MUX and electrodes was considered, and factors such as temperature-induced component variation or electrode interface changes during actual use were not fully incorporated. In particular, additional in vivo validation is required. Neurotransmitter concentrations in vivo are extremely low, and the ionic environment surrounding the electrode is highly complex. The quantitative effect of displacement current on biological signals must also be carefully assessed. Future work will include in vivo noise characterization, long-term multi-subject monitoring, and evaluation of signal variability under pharmacological or behavioral interventions.

The multichannel electrochemical system developed in this study is well-suited for neuroscience and pharmacology experiments that require simultaneous monitoring across multiple subjects to enhance statistical power and experimental throughput. The ability to apply custom waveforms also provides high flexibility for probing different neurotransmitters or drug conditions. These advantages extend to point-of-care diagnostics and therapeutic drug monitoring, where parallel measurements across multiple samples or dosing conditions are often required.

## Conclusion

In conclusion, this study provides a quantitative evaluation of the impact of MUX parasitic characteristics on electrochemical signal quality, offering design criteria tailored to electrochemical systems. These findings lay a technical foundation for developing reliable and scalable platforms for simultaneous neurotransmitter monitoring. Such platforms will advance electrochemical neural sensing technologies in neuroscience, pharmacology, point-of-care, and biofeedback applications.

## Supplementary Information

Below is the link to the electronic supplementary material.

Supplementary Material 1
