# Supplementary material for: Multiplexer-based system development for multichannel neurochemical sensing
Source: Biomed Eng Lett. 2026 Mar 2;16(4):929–43. doi: 10.1007/s13534-026-00554-3 (PMC13476222; doi:10.1007/s13534-026-00554-3)
Supplement: Supplementary file 1 [file 13534_2026_554_MOESM1_ESM.docx]

Supplementary Material

**Multiplexer-Based System Development
for Multichannel Neurochemical Sensing**

**Haeun Kwon^1ǂ^, Sang Mun Hwang^2^, Dong Pyo Jang^2*^**

^1^Department of Electronic Engineering, Hanyang University

^2^Department of Biomedical Engineering, Hanyang University

Figure S1. Effect of MUX ordering on voltage and current responses; Swapping the high-RC and low-RC MUX positions produced identical voltage and current outputs, confirming that the two-stage MUX configuration is structurally symmetric and that signal distortion depends only on the RC values, not on their ordering.

Figure S2. Frequency dependent effects of parasitic capacitance on voltage and current; Simulated FSCV responses were evaluated at 1, 2, 3, 5, 10, and 50 Hz to assess the influence of repeated switching frequency on waveform fidelity. (up) Raw and normalized voltage traces showed <0.5% variation across all conditions with no consistent capacitance-dependent trend. (down) Raw current amplitudes reflected the expected capacitance-dependent offset, but normalized currents varied by <0.5% across all frequencies. These results indicate that, within the typical operational range of FSCV, repetition frequency has a negligible effect on voltage propagation and current distortion, consistent with the large separation between the MUX RC time constants (nanoseconds) and FSCV cycle rates (Hz).

Figure S3. Scan rate dependent effects of parasitic capacitance on voltage and current; Simulations were conducted at scan rates from 100 to 5000 V/s to examine the effect of dV/dt on distortion. (up) As the scan rate increased, the voltage amplitude decreased linearly with larger reductions for higher parasitic capacitance. (down) The raw current increased in proportion to the scan rate, and this increase became more pronounced at higher capacitance. This behavior is consistent with the expected displacement-current contribution (I ≈ C·dV/dt). In contrast, the normalized current traces showed nearly identical slopes across capacitance conditions. This indicates that parasitic capacitance mainly shifts the baseline level, while the scan-rate-dependent change in current remains essentially unchanged.
